# Supplementary material for: Do government innovation preferences enhance ecological resilience in resource-based cities?—Based on mediating effect and threshold effect perspectives
Source: PLoS One. 2024 Nov 1;19(11):e0303672. doi: 10.1371/journal.pone.0303672 (PMC11530058; doi:10.1371/journal.pone.0303672)
Supplement: Attachment — Submitted filename: Response to Reviewers.docx [file pone.0303672.s003.docx]

**Response to Reviewers**

Dear Editors,

Thank you and the reviewing experts for your review comments. Based on your and the reviewers' comments, I have revised the manuscript, responding to each point raised one by one, as described below:

Journal Requirements:

1. The new manuscript meets the style requirements of PLOS ONE.

2. The raw data of this manuscript were uploaded in the first submission.

3. The code for this manuscript was uploaded as an attachment in this submission.

4. The funder's role in the study is stated in the new cover letter.

5. The ORCID IDs of the corresponding authors are provided in the new submission.

6. Fig.3 and Fig.5 have been deleted from the new manuscript. new table and figure have been used for the replacements.

7. TIFF/EPS image files are uploaded in the new manuscript.

8. Material related to supporting information is added at the end of the new manuscript.

Reviewer 1's comments:

1. Figure 1 of the original manuscript has been removed from the new manuscript, and only the section that discusses the structure of the article has been retained.

2. The new manuscript adds the contribution of this study to future research in the Introduction section and further refines the innovations of the article.

3. The new manuscript refines the reference citation of the statement and further explains the reason for using the threshold effect model.

4. The new manuscript refines the explanation for the results presented in the Discussion section.

5. This manuscript has been revised and embellished for language by native English-speaking professionals.

6. Owing to the literature citation suggestions given by the reviewers, the new manuscript cites the references that the reviewers suggested using in the article.

Reviewer 2's comments.

1. In the new manuscript, the Literature Review section has been rewritten so that it covers as much as possible all areas of this study. The reasons for the contribution to the literature have also been refined.

2. The new manuscript clearly states the research motivation, research questions and research contributions.

3. The new manuscript has been revised based on the format of PLOS ONE journals, especially the citation format, which has been reorganized and rearranged.

4. The new manuscript adds the theoretical and practical significance of this study and explains the conclusions in more depth.

5. The new manuscript eliminates most of the Chinese references and selects English-language references as a supplement.

6. The new manuscript has been revised and embellished by native English-speaking professionals.

To facilitate your re-review of the manuscript, the revisions responding to the comments regarding the journal requirements are annotated in green, the revisions responding to the comments of reviewer 1 are annotated in red, the revisions responding to the comments of reviewer 2 are annotated in blue, and the revisions responding to the comments of the two reviewers are annotated in violet. Thank you again for the valuable comments, and we hope that you will inform us if you find any other deficiencies in the process of the re-reviewing the manuscript. Thank you again.

Corresponding author: Jiahui Yang e-mail: [15136858260@163.com](mailto:15136858260@163.com).
